# *N* ^4^,*N* ^8^,3,6,9,10,11-Heptaphenyl-3,6,9,10,11-penta­azatricyclo­[5.2.1.1^2,5^]undecane-4,8-diamine

**DOI:** 10.1107/S1600536809034436

**Published:** 2009-09-05

**Authors:** Amir Taheri, Sayed Mojtaba Moosavi

**Affiliations:** a Department of Chemistry, Imam Hossein University, Tehran, Iran

## Abstract

The title compound, C_48_H_43_N_7_, is a polyaza­polycyclic compound with a near-*C*_2_ symmetric skeleton. In the crystal, a N—H⋯π inter­action occurs.

## Related literature

For the synthesis of the 2,5,7–triaza­bicyclo­[2.2.1]heptane derivative, see: Taheri & Moosavi (2009*a* ▶,*b* ▶). For general background to triaza­norbornanes, see: Nitravati & Sikhibhushan (1939 ▶). For the syntheses of polyaza­polycyclic compounds, see: Nielsen *et al.* (1990 ▶, 1992 ▶).
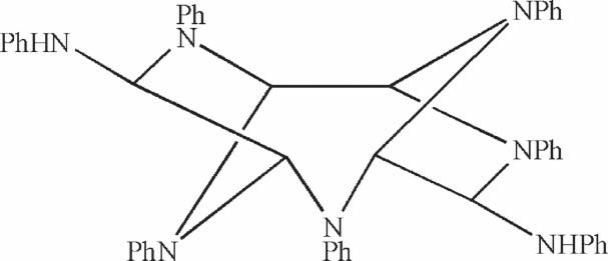


## Experimental

### 

#### Crystal data

C_48_H_43_N_7_
*M* *_r_* = 717.89
Triclinic, 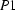

*a* = 9.4889 (19) Å
*b* = 9.6252 (19) Å
*c* = 10.967 (2) Å
α = 113.924 (4)°
β = 94.555 (4)°
γ = 93.835 (4)°
*V* = 907.3 (3) Å^3^
*Z* = 1
Mo *K*α radiation
μ = 0.08 mm^−1^
*T* = 100 K
0.28 × 0.15 × 0.06 mm

#### Data collection

Bruker APEXII CCD area-detector diffractometer
Absorption correction: none
9544 measured reflections
4343 independent reflections
2787 reflections with *I* > 2σ(*I*)
*R*_int_ = 0.062

#### Refinement

*R*[*F* ^2^ > 2σ(*F* ^2^)] = 0.056
*wR*(*F* ^2^) = 0.121
*S* = 1.03
4343 reflections
496 parameters
3 restraints
H-atom parameters constrained
Δρ_max_ = 0.25 e Å^−3^
Δρ_min_ = −0.24 e Å^−3^

### 

Data collection: *APEX2* (Bruker, 2005 ▶); cell refinement: *SAINT* (Bruker, 2005 ▶); data reduction: *SAINT* program(s) used to solve structure: *SHELXTL* (Sheldrick, 2008 ▶); program(s) used to refine structure: *SHELXTL*; molecular graphics: *SHELXTL*; software used to prepare material for publication: *SHELXTL*.

## Supplementary Material

Crystal structure: contains datablocks I, global. DOI: 10.1107/S1600536809034436/bx2238sup1.cif

Structure factors: contains datablocks I. DOI: 10.1107/S1600536809034436/bx2238Isup2.hkl

Additional supplementary materials:  crystallographic information; 3D view; checkCIF report

## Figures and Tables

**Table 1:** Hydrogen-bond geometry (Å, °)

*D*—H⋯*A*	*D*—H	H⋯*A*	*D*⋯*A*	*D*—H⋯*A*
N3—H3⋯*Cg*^i^	0.81	2.90	3.699 (4)	169

Symmetry code: (i) 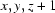. *Cg* is the centroid of the C71–C76 ring.

**Fig. 1.**
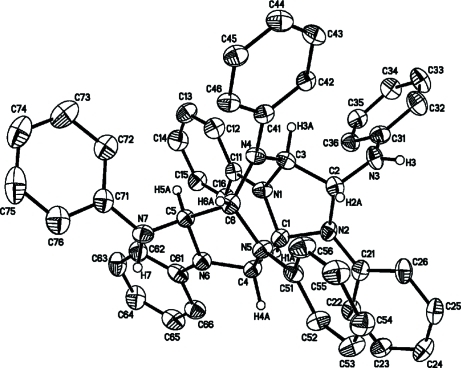
The molecular structure of the title compound, showing the atom-numbering scheme. Only H atoms bond to N and tertiary C atoms are shown. Displacement ellipsoids are at the 50% probability level.

## supplementary crystallographic information

### Comment

Polyazapolycyclic compounds are constituted of saturated ring systems with multiple N atoms with different kinds of skeletons which be utilized for the syntheses of other derivatives (Nielsen *et al.*, 1992). Among the cage skeletons, 2,4,6,8,10,12-Hexabenzyl-2,4,6,8,10,12-hexaazaisowurtzitane (HBIW) is a famous precursor for 2,4,6,8,10,12-Hexanitro-2,4,6,8,10,12-hexaazaisowurtzitane (HNIW), a highly energetic compound (Nielsen *et al.*, 1990). In the norbornane skeletons triazanorbornanes or triazabicyclo[2,2,1]heptanes have been determined (Nitravati & Sikhibhushan, 1939).

*N*^4^,*N*^8^,3,6,9,10,11-Heptaphenyl-3,6,9,10,11-pentaazatricyclo[5,2,1^1,7^,1^2,5^]undecane-4,8-diamine is synthesized for the first time *via* a catalytic reaction of *N*^3^,*N*^6^,2,5,7-pentaphenyl-2,5,7-triazabicyclo[2,2,1]heptane-3,6-diamine (Taheri & Mossavi, 2009*a*; Taheri & Mossavi, 2009*b*) with nickel(II)nitrate as reagent. An *ORTEP* diagram of the title compound is shown in Figure 1. It consists of a nine-membered ring and two N-atoms bridging between C1 and C3 as well as C4 and C6. The compound is chiral, but only the relative configuration of the chiral centres could be determined. The skeleton is almost C_2_ symmetric with a two-fold rotation axis running through the midpoint of the C1-C4 bond and N4.

There are no classical hydrogen bonds, just a weak intermolecular interactions, N3—H3···cg [(C71,C72, C73, C74, C75, C76) at *x*,*y*,*z* + *z*]: H···cg 2.90 Å, angle at H 168.6°.

### Experimental

To a stirred solution of *N*^3^,*N*^6^,2,5,7-pentaphenyl-2,5,7-triazabicyclo[2,2,1]heptane-\ 3,6-diamine(II) (1 mmol) in 20 ml of acetonitril was added 8.9 mg of nickel(II)nitrate (0.5 mmol) slowly at 288 K over 30 min. The reaction mixture was warmed up to 318 K and left for 40 min. The reaction mixture was then allowed to cool to 298 K and stand for 48 h. The precipitation was filtered and washed with cold ethanol to give a white powder 0.61 g (85% yield) of the title compound (m.p. 531 K). Recrystallization in hot dichloromethane yielded the single crystals for data collection. IR (KBr) (υ_max_/cm^-1^): 3358 (NH). ^1^H NMR (CDCl_3_): δ_H_ 6.74–7.24 (35*H*, m, CH_Ar_),6.30 (2*H*, s, CH), 5.56 (2*H*, s, CH), 5.21 (2*H*, d, ^2^*J*=7.0 Hz,NH), 3.89 (2*H*, d, ^2^*J*=7.0 Hz, NH).^13^C NMR (CDCl_3_):δ_C_ 146.9, 145.6, 145.2, 143.3, 142.5, 130.0, 129.7, 129.5, 129.4,129.1, 129.0, 119.9, 119.0, 118.8, 118.4, 118.3, 118.2, 116.3, 114.6, 114.3, 113.8,113.6, 113.5, 113.2 (CH_Ar_), 81.9 (2CH), 72.3 (2CH), 76.1 (2CH).

### Refinement

All H atoms were located in difference Fourier synthesis. They were refined using a riding model with N-H ranging from 0.81Å - 0.88 Å, C-H ranging from 0.95Å - 1.00 Å and with *U*_iso_(H) set to 1.2 *U*_eq_(C,N).

### Crystal data

**Table:**

C_48_H_43_N_7_	*Z* = 1
*M**_r_* = 717.89	*F*(000) = 380
Triclinic, *P*1	*D*_x_ = 1.314 Mg m^−^^3^
Hall symbol: P 1	Mo *K*α radiation, λ = 0.71073 Å
*a* = 9.4889 (19) Å	Cell parameters from 1186 reflections
*b* = 9.6252 (19) Å	θ = 2.3–22.0°
*c* = 10.967 (2) Å	µ = 0.08 mm^−^^1^
α = 113.924 (4)°	*T* = 100 K
β = 94.555 (4)°	Prism, red
γ = 93.835 (4)°	0.28 × 0.15 × 0.06 mm
*V* = 907.3 (3) Å^3^	

### Data collection

**Table:**

Bruker APEXII CCD area-detector diffractometer	2787 reflections with *I* > 2σ(*I*)
Radiation source: fine-focus sealed tube	*R*_int_ = 0.062
graphite	θ_max_ = 28.0°, θ_min_ = 2.1°
φ and ω scans	*h* = −12→12
9544 measured reflections	*k* = −12→12
4343 independent reflections	*l* = −14→14

### Refinement

**Table:**

Refinement on *F*^2^	Primary atom site location: structure-invariant direct methods
Least-squares matrix: full	Secondary atom site location: difference Fourier map
*R*[*F*^2^ > 2σ(*F*^2^)] = 0.056	Hydrogen site location: mixed
*wR*(*F*^2^) = 0.121	H-atom parameters constrained
*S* = 1.03	*w* = 1/[σ^2^(*F*_o_^2^) + (0.048*P*)^2^] where *P* = (*F*_o_^2^ + 2*F*_c_^2^)/3
4343 reflections	(Δ/σ)_max_ < 0.001
496 parameters	Δρ_max_ = 0.25 e Å^−^^3^
3 restraints	Δρ_min_ = −0.24 e Å^−^^3^

### Special details

**Table:**

Geometry. All e.s.d.'s (except the e.s.d. in the dihedral angle between two l.s. planes) are estimated using the full covariance matrix. The cell e.s.d.'s are taken into account individually in the estimation of e.s.d.'s in distances, angles and torsion angles; correlations between e.s.d.'s in cell parameters are only used when they are defined by crystal symmetry. An approximate (isotropic) treatment of cell e.s.d.'s is used for estimating e.s.d.'s involving l.s. planes.
Refinement. Refinement of *F*^2^ against ALL reflections. The weighted *R*-factor *wR* and goodness of fit *S* are based on *F*^2^, conventional *R*-factors *R* are based on *F*, with *F* set to zero for negative *F*^2^. The threshold expression of *F*^2^ > σ(*F*^2^) is used only for calculating *R*-factors(gt) *etc*. and is not relevant to the choice of reflections for refinement. *R*-factors based on *F*^2^ are statistically about twice as large as those based on *F*, and *R*- factors based on ALL data will be even larger.

### Fractional atomic coordinates and isotropic or equivalent isotropic displacement parameters (Å^2^)

**Table:**

	*x*	*y*	*z*	*U*_iso_*/*U*_eq_	
N1	−0.1525 (3)	0.6552 (4)	0.2565 (3)	0.0243 (8)	
N2	−0.0946 (3)	0.7828 (4)	0.4788 (3)	0.0251 (8)	
N3	−0.0754 (4)	0.5526 (4)	0.5139 (4)	0.0310 (9)	
H3	−0.0231	0.5589	0.5787	0.037*	
N4	0.0672 (3)	0.5451 (4)	0.2164 (3)	0.0232 (7)	
N5	0.1312 (3)	0.8199 (4)	0.3351 (3)	0.0233 (8)	
N6	−0.0103 (3)	0.8702 (4)	0.1880 (3)	0.0249 (8)	
N7	0.1492 (3)	0.7731 (4)	0.0288 (3)	0.0272 (8)	
H7	0.1815	0.8690	0.0534	0.033*	
C1	−0.1258 (4)	0.8074 (5)	0.3592 (4)	0.0255 (9)	
H1A	−0.2120	0.8629	0.3646	0.031*	
C2	−0.0255 (4)	0.6409 (5)	0.4445 (4)	0.0259 (10)	
H2A	0.0796	0.6679	0.4686	0.031*	
C3	−0.0588 (4)	0.5602 (5)	0.2896 (4)	0.0238 (9)	
H3A	−0.1106	0.4566	0.2639	0.029*	
C4	0.0039 (4)	0.8930 (4)	0.3268 (4)	0.0225 (9)	
H4A	0.0175	1.0042	0.3883	0.027*	
C5	0.0517 (4)	0.7329 (5)	0.1059 (4)	0.0223 (9)	
H5A	−0.0238	0.6504	0.0462	0.027*	
C6	0.1311 (4)	0.6857 (5)	0.2101 (4)	0.0255 (9)	
H6A	0.2315	0.6725	0.1897	0.031*	
C11	−0.2637 (4)	0.5993 (5)	0.1503 (4)	0.0233 (9)	
C12	−0.2673 (4)	0.4571 (5)	0.0480 (5)	0.0331 (11)	
H12A	−0.1955	0.3938	0.0506	0.040*	
C13	−0.3732 (4)	0.4043 (5)	−0.0586 (5)	0.0353 (11)	
H13A	−0.3741	0.3049	−0.1282	0.042*	
C14	−0.4789 (5)	0.4956 (5)	−0.0651 (5)	0.0381 (12)	
H14A	−0.5501	0.4616	−0.1400	0.046*	
C15	−0.4775 (5)	0.6360 (5)	0.0396 (4)	0.0325 (11)	
H15A	−0.5502	0.6985	0.0374	0.039*	
C16	−0.3740 (4)	0.6886 (5)	0.1473 (4)	0.0261 (10)	
H16A	−0.3768	0.7851	0.2196	0.031*	
C21	−0.0692 (4)	0.9047 (5)	0.6055 (4)	0.0267 (9)	
C22	−0.1324 (4)	1.0411 (5)	0.6308 (4)	0.0298 (10)	
H22A	−0.1922	1.0497	0.5611	0.036*	
C23	−0.1084 (5)	1.1597 (5)	0.7535 (5)	0.0343 (11)	
H23A	−0.1507	1.2510	0.7679	0.041*	
C24	−0.0243 (5)	1.1509 (6)	0.8581 (5)	0.0395 (12)	
H24A	−0.0100	1.2340	0.9442	0.047*	
C25	0.0386 (5)	1.0193 (6)	0.8349 (4)	0.0361 (11)	
H25A	0.0988	1.0126	0.9054	0.043*	
C26	0.0159 (5)	0.8977 (6)	0.7122 (4)	0.0341 (11)	
H26A	0.0587	0.8071	0.6993	0.041*	
C31	−0.2083 (4)	0.4805 (5)	0.5026 (5)	0.0300 (10)	
C32	−0.2259 (5)	0.3798 (5)	0.5641 (5)	0.0390 (12)	
H32A	−0.1475	0.3667	0.6164	0.047*	
C33	−0.3554 (5)	0.2998 (6)	0.5492 (5)	0.0415 (13)	
H33A	−0.3647	0.2282	0.5882	0.050*	
C34	−0.4730 (5)	0.3211 (5)	0.4788 (5)	0.0374 (11)	
H34A	−0.5627	0.2657	0.4699	0.045*	
C35	−0.4578 (4)	0.4236 (5)	0.4217 (5)	0.0331 (11)	
H35A	−0.5386	0.4400	0.3742	0.040*	
C36	−0.3283 (4)	0.5035 (5)	0.4315 (5)	0.0313 (10)	
H36A	−0.3200	0.5735	0.3907	0.038*	
C41	0.1493 (4)	0.4233 (4)	0.1994 (4)	0.0236 (9)	
C42	0.1242 (4)	0.3272 (4)	0.2642 (4)	0.0251 (9)	
H42A	0.0537	0.3464	0.3242	0.030*	
C43	0.2018 (4)	0.2039 (5)	0.2412 (5)	0.0300 (10)	
H43A	0.1817	0.1373	0.2836	0.036*	
C44	0.3079 (4)	0.1759 (5)	0.1577 (5)	0.0331 (11)	
H44A	0.3619	0.0923	0.1441	0.040*	
C45	0.3341 (4)	0.2711 (5)	0.0946 (5)	0.0306 (10)	
H45A	0.4066	0.2525	0.0367	0.037*	
C46	0.2562 (4)	0.3936 (4)	0.1141 (4)	0.0268 (9)	
H46A	0.2756	0.4579	0.0694	0.032*	
C51	0.2448 (4)	0.8775 (5)	0.4368 (4)	0.0248 (9)	
C52	0.2463 (4)	1.0172 (5)	0.5463 (4)	0.0294 (10)	
H52A	0.1686	1.0761	0.5523	0.035*	
C53	0.3605 (5)	1.0712 (5)	0.6467 (5)	0.0347 (11)	
H53A	0.3592	1.1662	0.7213	0.042*	
C54	0.4758 (5)	0.9894 (5)	0.6402 (5)	0.0336 (11)	
H54A	0.5545	1.0278	0.7084	0.040*	
C55	0.4739 (5)	0.8506 (5)	0.5322 (5)	0.0357 (11)	
H55A	0.5525	0.7930	0.5264	0.043*	
C56	0.3609 (4)	0.7933 (5)	0.4323 (5)	0.0341 (11)	
H56A	0.3616	0.6962	0.3600	0.041*	
C61	−0.1228 (4)	0.9217 (5)	0.1294 (5)	0.0286 (10)	
C62	−0.1656 (4)	0.8543 (5)	−0.0059 (5)	0.0313 (10)	
H62A	−0.1200	0.7705	−0.0621	0.038*	
C63	−0.2761 (5)	0.9077 (6)	−0.0627 (5)	0.0368 (11)	
H63A	−0.3052	0.8600	−0.1567	0.044*	
C64	−0.3418 (5)	1.0297 (6)	0.0188 (5)	0.0391 (12)	
H64A	−0.4172	1.0657	−0.0186	0.047*	
C65	−0.2976 (5)	1.0993 (5)	0.1552 (5)	0.0375 (12)	
H65A	−0.3431	1.1833	0.2110	0.045*	
C66	−0.1882 (4)	1.0486 (5)	0.2114 (5)	0.0329 (11)	
H66A	−0.1573	1.0990	0.3048	0.039*	
C71	0.1765 (4)	0.6732 (5)	−0.0973 (4)	0.0290 (10)	
C72	0.1245 (4)	0.5187 (5)	−0.1531 (5)	0.0356 (11)	
H72A	0.0688	0.4791	−0.1038	0.043*	
C73	0.1538 (5)	0.4225 (6)	−0.2802 (5)	0.0449 (13)	
H73A	0.1165	0.3179	−0.3174	0.054*	
C74	0.2338 (5)	0.4747 (7)	−0.3516 (5)	0.0458 (13)	
H74A	0.2502	0.4080	−0.4397	0.055*	
C75	0.2917 (5)	0.6242 (7)	−0.2973 (5)	0.0491 (14)	
H75A	0.3515	0.6596	−0.3465	0.059*	
C76	0.2640 (5)	0.7246 (6)	−0.1709 (5)	0.0401 (12)	
H76A	0.3044	0.8282	−0.1344	0.048*	

### Atomic displacement parameters (Å^2^)

**Table:**

	*U*^11^	*U*^22^	*U*^33^	*U*^12^	*U*^13^	*U*^23^
N1	0.0191 (16)	0.0197 (18)	0.031 (2)	0.0057 (14)	−0.0021 (15)	0.0081 (16)
N2	0.0234 (18)	0.030 (2)	0.0216 (19)	0.0074 (15)	0.0003 (15)	0.0098 (17)
N3	0.0276 (19)	0.041 (2)	0.033 (2)	0.0065 (16)	−0.0004 (16)	0.0245 (18)
N4	0.0186 (17)	0.0204 (17)	0.031 (2)	0.0054 (14)	0.0036 (14)	0.0106 (15)
N5	0.0195 (17)	0.0219 (19)	0.025 (2)	0.0091 (14)	0.0004 (15)	0.0056 (16)
N6	0.0239 (17)	0.0233 (19)	0.028 (2)	0.0095 (14)	0.0032 (15)	0.0106 (16)
N7	0.0257 (19)	0.0223 (19)	0.033 (2)	0.0075 (15)	0.0063 (16)	0.0102 (17)
C1	0.024 (2)	0.026 (2)	0.025 (2)	0.0081 (17)	0.0009 (18)	0.0081 (19)
C2	0.021 (2)	0.032 (2)	0.030 (3)	0.0084 (17)	0.0045 (18)	0.016 (2)
C3	0.0173 (19)	0.024 (2)	0.034 (2)	0.0053 (16)	0.0023 (18)	0.0144 (19)
C4	0.021 (2)	0.021 (2)	0.023 (2)	0.0075 (16)	−0.0003 (17)	0.0063 (18)
C5	0.022 (2)	0.022 (2)	0.024 (2)	0.0079 (16)	0.0002 (18)	0.0098 (18)
C6	0.023 (2)	0.027 (2)	0.025 (2)	0.0052 (17)	0.0029 (18)	0.0095 (19)
C11	0.018 (2)	0.028 (2)	0.025 (2)	0.0025 (17)	0.0008 (17)	0.0120 (19)
C12	0.025 (2)	0.036 (3)	0.036 (3)	0.0055 (19)	−0.001 (2)	0.013 (2)
C13	0.030 (2)	0.036 (3)	0.032 (3)	0.003 (2)	−0.001 (2)	0.006 (2)
C14	0.031 (2)	0.041 (3)	0.040 (3)	0.001 (2)	−0.007 (2)	0.016 (2)
C15	0.028 (2)	0.036 (3)	0.032 (3)	0.0084 (19)	−0.001 (2)	0.013 (2)
C16	0.021 (2)	0.028 (2)	0.027 (2)	0.0072 (17)	0.0019 (19)	0.009 (2)
C21	0.019 (2)	0.034 (2)	0.026 (2)	−0.0017 (18)	0.0021 (17)	0.012 (2)
C22	0.024 (2)	0.035 (3)	0.032 (3)	0.0085 (19)	0.0004 (19)	0.014 (2)
C23	0.036 (3)	0.028 (3)	0.033 (3)	0.0059 (19)	0.004 (2)	0.006 (2)
C24	0.041 (3)	0.042 (3)	0.025 (3)	0.005 (2)	0.003 (2)	0.003 (2)
C25	0.034 (3)	0.051 (3)	0.026 (3)	0.004 (2)	0.001 (2)	0.019 (2)
C26	0.032 (2)	0.046 (3)	0.029 (3)	0.008 (2)	−0.001 (2)	0.021 (2)
C31	0.027 (2)	0.026 (2)	0.035 (3)	0.0078 (18)	0.004 (2)	0.010 (2)
C32	0.031 (2)	0.047 (3)	0.048 (3)	0.007 (2)	0.005 (2)	0.030 (3)
C33	0.035 (3)	0.052 (3)	0.053 (3)	0.003 (2)	0.006 (2)	0.037 (3)
C34	0.027 (2)	0.041 (3)	0.046 (3)	0.002 (2)	0.004 (2)	0.020 (2)
C35	0.026 (2)	0.034 (3)	0.036 (3)	0.0058 (19)	0.000 (2)	0.011 (2)
C36	0.032 (2)	0.029 (2)	0.034 (3)	0.0075 (19)	0.005 (2)	0.014 (2)
C41	0.018 (2)	0.024 (2)	0.025 (2)	0.0012 (16)	−0.0026 (17)	0.0072 (18)
C42	0.022 (2)	0.026 (2)	0.025 (2)	0.0052 (17)	−0.0030 (18)	0.0090 (19)
C43	0.027 (2)	0.026 (2)	0.040 (3)	0.0035 (18)	0.000 (2)	0.017 (2)
C44	0.025 (2)	0.029 (2)	0.045 (3)	0.0117 (19)	0.003 (2)	0.014 (2)
C45	0.026 (2)	0.028 (2)	0.036 (3)	0.0111 (19)	0.007 (2)	0.010 (2)
C46	0.022 (2)	0.022 (2)	0.034 (3)	0.0024 (17)	−0.0007 (19)	0.0103 (19)
C51	0.022 (2)	0.025 (2)	0.030 (2)	0.0031 (17)	0.0013 (18)	0.014 (2)
C52	0.024 (2)	0.025 (2)	0.032 (3)	0.0014 (17)	0.003 (2)	0.005 (2)
C53	0.030 (2)	0.027 (2)	0.036 (3)	0.0007 (19)	0.000 (2)	0.003 (2)
C54	0.026 (2)	0.034 (3)	0.036 (3)	−0.0005 (19)	−0.008 (2)	0.012 (2)
C55	0.028 (2)	0.032 (3)	0.045 (3)	0.0086 (19)	−0.002 (2)	0.014 (2)
C56	0.029 (2)	0.026 (2)	0.041 (3)	0.0068 (19)	−0.002 (2)	0.007 (2)
C61	0.022 (2)	0.028 (2)	0.043 (3)	0.0075 (17)	0.0059 (19)	0.021 (2)
C62	0.034 (2)	0.032 (2)	0.033 (3)	0.0144 (19)	0.010 (2)	0.016 (2)
C63	0.035 (3)	0.050 (3)	0.034 (3)	0.015 (2)	0.002 (2)	0.024 (2)
C64	0.035 (3)	0.048 (3)	0.042 (3)	0.022 (2)	0.008 (2)	0.024 (3)
C65	0.032 (2)	0.032 (3)	0.057 (3)	0.023 (2)	0.015 (2)	0.023 (2)
C66	0.032 (2)	0.027 (2)	0.041 (3)	0.0095 (19)	0.010 (2)	0.013 (2)
C71	0.023 (2)	0.037 (3)	0.030 (2)	0.0085 (18)	0.0001 (19)	0.017 (2)
C72	0.025 (2)	0.043 (3)	0.039 (3)	0.011 (2)	0.005 (2)	0.015 (2)
C73	0.031 (3)	0.050 (3)	0.041 (3)	0.015 (2)	−0.001 (2)	0.005 (3)
C74	0.035 (3)	0.066 (4)	0.033 (3)	0.022 (3)	0.005 (2)	0.014 (3)
C75	0.034 (3)	0.087 (4)	0.041 (3)	0.026 (3)	0.013 (2)	0.038 (3)
C76	0.034 (3)	0.050 (3)	0.042 (3)	0.013 (2)	0.003 (2)	0.024 (3)

### Geometric parameters (Å, °)

**Table:**

N1—C11	1.407 (5)	C32—C33	1.367 (6)
N1—C1	1.430 (5)	C32—H32A	0.9500
N1—C3	1.444 (5)	C33—C34	1.380 (6)
N2—C21	1.397 (5)	C33—H33A	0.9500
N2—C1	1.437 (5)	C34—C35	1.373 (6)
N2—C2	1.476 (5)	C34—H34A	0.9500
N3—C31	1.370 (5)	C35—C36	1.377 (6)
N3—C2	1.433 (5)	C35—H35A	0.9500
N3—H3	0.8127	C36—H36A	0.9500
N4—C41	1.409 (5)	C41—C42	1.396 (6)
N4—C3	1.475 (5)	C41—C46	1.399 (6)
N4—C6	1.475 (5)	C42—C43	1.385 (6)
N5—C51	1.394 (5)	C42—H42A	0.9500
N5—C6	1.453 (5)	C43—C44	1.381 (6)
N5—C4	1.453 (5)	C43—H43A	0.9500
N6—C61	1.421 (5)	C44—C45	1.376 (6)
N6—C4	1.441 (5)	C44—H44A	0.9500
N6—C5	1.458 (5)	C45—C46	1.385 (5)
N7—C71	1.384 (5)	C45—H45A	0.9500
N7—C5	1.436 (5)	C46—H46A	0.9500
N7—H7	0.8756	C51—C52	1.391 (6)
C1—C4	1.577 (6)	C51—C56	1.403 (5)
C1—H1A	1.0000	C52—C53	1.389 (6)
C2—C3	1.550 (6)	C52—H52A	0.9500
C2—H2A	1.0000	C53—C54	1.380 (6)
C3—H3A	1.0000	C53—H53A	0.9500
C4—H4A	1.0000	C54—C55	1.378 (6)
C5—C6	1.552 (6)	C54—H54A	0.9500
C5—H5A	1.0000	C55—C56	1.377 (6)
C6—H6A	1.0000	C55—H55A	0.9500
C11—C12	1.370 (6)	C56—H56A	0.9500
C11—C16	1.404 (5)	C61—C62	1.369 (6)
C12—C13	1.378 (6)	C61—C66	1.409 (6)
C12—H12A	0.9500	C62—C63	1.407 (6)
C13—C14	1.394 (6)	C62—H62A	0.9500
C13—H13A	0.9500	C63—C64	1.379 (6)
C14—C15	1.371 (6)	C63—H63A	0.9500
C14—H14A	0.9500	C64—C65	1.383 (7)
C15—C16	1.371 (6)	C64—H64A	0.9500
C15—H15A	0.9500	C65—C66	1.380 (6)
C16—H16A	0.9500	C65—H65A	0.9500
C21—C26	1.395 (6)	C66—H66A	0.9500
C21—C22	1.412 (6)	C71—C72	1.394 (6)
C22—C23	1.355 (6)	C71—C76	1.400 (6)
C22—H22A	0.9500	C72—C73	1.389 (7)
C23—C24	1.379 (6)	C72—H72A	0.9500
C23—H23A	0.9500	C73—C74	1.343 (7)
C24—C25	1.374 (7)	C73—H73A	0.9500
C24—H24A	0.9500	C74—C75	1.369 (8)
C25—C26	1.368 (6)	C74—H74A	0.9500
C25—H25A	0.9500	C75—C76	1.389 (7)
C26—H26A	0.9500	C75—H75A	0.9500
C31—C32	1.396 (6)	C76—H76A	0.9500
C31—C36	1.409 (6)		
			
C11—N1—C1	126.6 (3)	C21—C26—H26A	119.5
C11—N1—C3	124.2 (3)	N3—C31—C32	118.7 (4)
C1—N1—C3	108.7 (3)	N3—C31—C36	122.9 (4)
C21—N2—C1	121.5 (3)	C32—C31—C36	118.5 (4)
C21—N2—C2	122.7 (3)	C33—C32—C31	120.3 (4)
C1—N2—C2	109.0 (3)	C33—C32—H32A	119.8
C31—N3—C2	129.2 (4)	C31—C32—H32A	119.8
C31—N3—H3	113.7	C32—C33—C34	121.3 (5)
C2—N3—H3	115.9	C32—C33—H33A	119.3
C41—N4—C3	119.6 (3)	C34—C33—H33A	119.3
C41—N4—C6	120.0 (3)	C35—C34—C33	118.7 (4)
C3—N4—C6	116.3 (3)	C35—C34—H34A	120.6
C51—N5—C6	124.4 (3)	C33—C34—H34A	120.6
C51—N5—C4	126.5 (3)	C34—C35—C36	121.7 (4)
C6—N5—C4	108.6 (3)	C34—C35—H35A	119.2
C61—N6—C4	123.0 (3)	C36—C35—H35A	119.2
C61—N6—C5	119.8 (3)	C35—C36—C31	119.4 (4)
C4—N6—C5	110.7 (3)	C35—C36—H36A	120.3
C71—N7—C5	123.5 (3)	C31—C36—H36A	120.3
C71—N7—H7	115.8	C42—C41—C46	118.3 (4)
C5—N7—H7	119.6	C42—C41—N4	121.1 (4)
N1—C1—N2	102.8 (3)	C46—C41—N4	120.6 (4)
N1—C1—C4	108.6 (3)	C43—C42—C41	120.2 (4)
N2—C1—C4	113.0 (3)	C43—C42—H42A	119.9
N1—C1—H1A	110.7	C41—C42—H42A	119.9
N2—C1—H1A	110.7	C44—C43—C42	121.2 (4)
C4—C1—H1A	110.7	C44—C43—H43A	119.4
N3—C2—N2	112.1 (3)	C42—C43—H43A	119.4
N3—C2—C3	114.4 (3)	C45—C44—C43	118.9 (4)
N2—C2—C3	103.1 (3)	C45—C44—H44A	120.6
N3—C2—H2A	109.0	C43—C44—H44A	120.6
N2—C2—H2A	109.0	C44—C45—C46	121.0 (4)
C3—C2—H2A	109.0	C44—C45—H45A	119.5
N1—C3—N4	110.1 (3)	C46—C45—H45A	119.5
N1—C3—C2	104.1 (3)	C45—C46—C41	120.4 (4)
N4—C3—C2	114.5 (3)	C45—C46—H46A	119.8
N1—C3—H3A	109.3	C41—C46—H46A	119.8
N4—C3—H3A	109.3	C52—C51—N5	121.7 (4)
C2—C3—H3A	109.3	C52—C51—C56	118.0 (4)
N6—C4—N5	101.4 (3)	N5—C51—C56	120.3 (4)
N6—C4—C1	111.7 (3)	C53—C52—C51	120.5 (4)
N5—C4—C1	109.3 (3)	C53—C52—H52A	119.8
N6—C4—H4A	111.3	C51—C52—H52A	119.8
N5—C4—H4A	111.3	C54—C53—C52	121.3 (4)
C1—C4—H4A	111.3	C54—C53—H53A	119.4
N7—C5—N6	108.2 (3)	C52—C53—H53A	119.4
N7—C5—C6	111.1 (3)	C55—C54—C53	118.2 (4)
N6—C5—C6	104.0 (3)	C55—C54—H54A	120.9
N7—C5—H5A	111.1	C53—C54—H54A	120.9
N6—C5—H5A	111.1	C56—C55—C54	121.7 (4)
C6—C5—H5A	111.1	C56—C55—H55A	119.1
N5—C6—N4	112.4 (3)	C54—C55—H55A	119.1
N5—C6—C5	102.5 (3)	C55—C56—C51	120.3 (4)
N4—C6—C5	115.3 (3)	C55—C56—H56A	119.8
N5—C6—H6A	108.8	C51—C56—H56A	119.8
N4—C6—H6A	108.8	C62—C61—C66	119.2 (4)
C5—C6—H6A	108.8	C62—C61—N6	121.4 (4)
C12—C11—C16	118.5 (4)	C66—C61—N6	119.3 (4)
C12—C11—N1	121.0 (3)	C61—C62—C63	120.8 (4)
C16—C11—N1	120.5 (4)	C61—C62—H62A	119.6
C11—C12—C13	121.2 (4)	C63—C62—H62A	119.6
C11—C12—H12A	119.4	C64—C63—C62	119.6 (4)
C13—C12—H12A	119.4	C64—C63—H63A	120.2
C12—C13—C14	120.4 (4)	C62—C63—H63A	120.2
C12—C13—H13A	119.8	C63—C64—C65	119.8 (4)
C14—C13—H13A	119.8	C63—C64—H64A	120.1
C15—C14—C13	118.2 (4)	C65—C64—H64A	120.1
C15—C14—H14A	120.9	C66—C65—C64	121.0 (4)
C13—C14—H14A	120.9	C66—C65—H65A	119.5
C16—C15—C14	121.8 (4)	C64—C65—H65A	119.5
C16—C15—H15A	119.1	C65—C66—C61	119.7 (4)
C14—C15—H15A	119.1	C65—C66—H66A	120.2
C15—C16—C11	119.8 (4)	C61—C66—H66A	120.2
C15—C16—H16A	120.1	N7—C71—C72	122.2 (4)
C11—C16—H16A	120.1	N7—C71—C76	120.1 (4)
C26—C21—N2	122.6 (4)	C72—C71—C76	117.7 (4)
C26—C21—C22	117.1 (4)	C73—C72—C71	120.3 (5)
N2—C21—C22	120.3 (4)	C73—C72—H72A	119.8
C23—C22—C21	120.7 (4)	C71—C72—H72A	119.8
C23—C22—H22A	119.7	C74—C73—C72	121.2 (5)
C21—C22—H22A	119.7	C74—C73—H73A	119.4
C22—C23—C24	121.5 (4)	C72—C73—H73A	119.4
C22—C23—H23A	119.2	C73—C74—C75	119.8 (5)
C24—C23—H23A	119.2	C73—C74—H74A	120.1
C25—C24—C23	118.5 (4)	C75—C74—H74A	120.1
C25—C24—H24A	120.7	C74—C75—C76	120.8 (5)
C23—C24—H24A	120.7	C74—C75—H75A	119.6
C26—C25—C24	121.1 (4)	C76—C75—H75A	119.6
C26—C25—H25A	119.4	C75—C76—C71	120.0 (5)
C24—C25—H25A	119.4	C75—C76—H76A	120.0
C25—C26—C21	121.0 (4)	C71—C76—H76A	120.0
C25—C26—H26A	119.5		
			
C11—N1—C1—N2	136.1 (4)	C1—N2—C21—C26	−152.4 (4)
C3—N1—C1—N2	−35.8 (4)	C2—N2—C21—C26	−4.5 (6)
C11—N1—C1—C4	−104.0 (4)	C1—N2—C21—C22	28.7 (5)
C3—N1—C1—C4	84.2 (4)	C2—N2—C21—C22	176.6 (4)
C21—N2—C1—N1	−176.5 (3)	C26—C21—C22—C23	0.9 (6)
C2—N2—C1—N1	31.7 (4)	N2—C21—C22—C23	179.9 (4)
C21—N2—C1—C4	66.6 (4)	C21—C22—C23—C24	−1.1 (7)
C2—N2—C1—C4	−85.1 (4)	C22—C23—C24—C25	1.4 (7)
C31—N3—C2—N2	65.4 (6)	C23—C24—C25—C26	−1.6 (7)
C31—N3—C2—C3	−51.5 (6)	C24—C25—C26—C21	1.5 (7)
C21—N2—C2—N3	69.0 (5)	N2—C21—C26—C25	180.0 (4)
C1—N2—C2—N3	−139.6 (3)	C22—C21—C26—C25	−1.1 (6)
C21—N2—C2—C3	−167.5 (3)	C2—N3—C31—C32	169.3 (4)
C1—N2—C2—C3	−16.1 (4)	C2—N3—C31—C36	−10.7 (7)
C11—N1—C3—N4	90.5 (4)	N3—C31—C32—C33	−176.7 (4)
C1—N1—C3—N4	−97.4 (4)	C36—C31—C32—C33	3.3 (7)
C11—N1—C3—C2	−146.3 (4)	C31—C32—C33—C34	−2.9 (8)
C1—N1—C3—C2	25.8 (4)	C32—C33—C34—C35	0.7 (7)
C41—N4—C3—N1	−160.3 (3)	C33—C34—C35—C36	1.0 (7)
C6—N4—C3—N1	42.2 (4)	C34—C35—C36—C31	−0.4 (7)
C41—N4—C3—C2	82.8 (4)	N3—C31—C36—C35	178.3 (4)
C6—N4—C3—C2	−74.7 (4)	C32—C31—C36—C35	−1.7 (6)
N3—C2—C3—N1	116.4 (3)	C3—N4—C41—C42	−7.9 (5)
N2—C2—C3—N1	−5.6 (4)	C6—N4—C41—C42	148.8 (4)
N3—C2—C3—N4	−123.4 (4)	C3—N4—C41—C46	170.6 (4)
N2—C2—C3—N4	114.7 (4)	C6—N4—C41—C46	−32.7 (5)
C61—N6—C4—N5	179.4 (3)	C46—C41—C42—C43	−1.5 (6)
C5—N6—C4—N5	28.0 (4)	N4—C41—C42—C43	177.0 (4)
C61—N6—C4—C1	63.0 (5)	C41—C42—C43—C44	2.0 (6)
C5—N6—C4—C1	−88.4 (4)	C42—C43—C44—C45	−1.4 (7)
C51—N5—C4—N6	135.2 (4)	C43—C44—C45—C46	0.2 (7)
C6—N5—C4—N6	−36.8 (4)	C44—C45—C46—C41	0.2 (6)
C51—N5—C4—C1	−106.7 (4)	C42—C41—C46—C45	0.4 (6)
C6—N5—C4—C1	81.4 (4)	N4—C41—C46—C45	−178.1 (4)
N1—C1—C4—N6	46.6 (4)	C6—N5—C51—C52	171.4 (4)
N2—C1—C4—N6	160.0 (3)	C4—N5—C51—C52	0.6 (6)
N1—C1—C4—N5	−64.9 (4)	C6—N5—C51—C56	−9.8 (6)
N2—C1—C4—N5	48.5 (4)	C4—N5—C51—C56	179.4 (4)
C71—N7—C5—N6	−149.1 (3)	N5—C51—C52—C53	179.5 (4)
C71—N7—C5—C6	97.3 (4)	C56—C51—C52—C53	0.6 (6)
C61—N6—C5—N7	79.5 (4)	C51—C52—C53—C54	0.8 (7)
C4—N6—C5—N7	−128.0 (3)	C52—C53—C54—C55	−1.2 (7)
C61—N6—C5—C6	−162.3 (3)	C53—C54—C55—C56	0.1 (7)
C4—N6—C5—C6	−9.8 (4)	C54—C55—C56—C51	1.4 (7)
C51—N5—C6—N4	94.4 (4)	C52—C51—C56—C55	−1.7 (7)
C4—N5—C6—N4	−93.4 (4)	N5—C51—C56—C55	179.5 (4)
C51—N5—C6—C5	−141.3 (4)	C4—N6—C61—C62	−155.6 (4)
C4—N5—C6—C5	30.9 (4)	C5—N6—C61—C62	−6.6 (6)
C41—N4—C6—N5	−118.5 (4)	C4—N6—C61—C66	26.9 (6)
C3—N4—C6—N5	38.9 (5)	C5—N6—C61—C66	175.9 (4)
C41—N4—C6—C5	124.6 (4)	C66—C61—C62—C63	−1.7 (6)
C3—N4—C6—C5	−78.0 (4)	N6—C61—C62—C63	−179.3 (4)
N7—C5—C6—N5	103.7 (4)	C61—C62—C63—C64	0.2 (7)
N6—C5—C6—N5	−12.5 (4)	C62—C63—C64—C65	0.7 (7)
N7—C5—C6—N4	−133.9 (3)	C63—C64—C65—C66	0.0 (7)
N6—C5—C6—N4	109.9 (4)	C64—C65—C66—C61	−1.6 (7)
C1—N1—C11—C12	171.1 (4)	C62—C61—C66—C65	2.4 (6)
C3—N1—C11—C12	−18.2 (6)	N6—C61—C66—C65	180.0 (4)
C1—N1—C11—C16	−9.0 (6)	C5—N7—C71—C72	−8.1 (6)
C3—N1—C11—C16	161.6 (4)	C5—N7—C71—C76	174.7 (4)
C16—C11—C12—C13	2.5 (7)	N7—C71—C72—C73	179.6 (4)
N1—C11—C12—C13	−177.6 (4)	C76—C71—C72—C73	−3.1 (6)
C11—C12—C13—C14	0.5 (7)	C71—C72—C73—C74	0.9 (7)
C12—C13—C14—C15	−2.5 (7)	C72—C73—C74—C75	2.0 (7)
C13—C14—C15—C16	1.4 (7)	C73—C74—C75—C76	−2.6 (7)
C14—C15—C16—C11	1.6 (7)	C74—C75—C76—C71	0.3 (7)
C12—C11—C16—C15	−3.6 (6)	N7—C71—C76—C75	179.8 (4)
N1—C11—C16—C15	176.5 (4)	C72—C71—C76—C75	2.5 (6)

### Hydrogen-bond geometry (Å, °)

**Table:**

*D*—H···*A*	*D*—H	H···*A*	*D*···*A*	*D*—H···*A*
N3—H3···Cg^i^	0.81	2.90	3.699 (4)	169

Symmetry codes: (i) *x*, *y*, *z*+1.
